# Antibody response of healthy children to pandemic A/H1N1/2009 influenza virus

**DOI:** 10.1186/1743-422X-8-563

**Published:** 2011-12-30

**Authors:** Susanna Esposito, Cristina Daleno, Claudia Tagliabue, Alessia Scala, Irene Picciolli, Francesca Taroni, Carlotta Galeone, Fausto Baldanti, Nicola Principi

**Affiliations:** 1 Department of Maternal and Pediatric Sciences, Università degli Studi di Milano, Fondazione IRCCS Ca' Granda Ospedale Maggiore Policlinico, Via Commenda 9, 20122 Milan, Italy; 2 Department of Epidemiology, Istituto di Ricerche Farmacologiche Mario Negri, Milan, Italy; 3 Department of Occupational Health "Luigi Devoto", University of Milan, Milan, Italy; 4 Molecular Virology Unit, Fondazione IRCCS Policlinico San Matteo, Pavia, Italy

**Keywords:** Children, Immune response, Influenza, Pandemic A/H1N1/2009 influenza virus, Pediatric infectious diseases

## Abstract

**Background:**

Little is known about the proportion of pediatric pandemic A/H1N1/2009 influenza cases who showed seroconversion, the magnitude of this seroconversion, or the factors that can affect the antibody level evoked by the pandemic A/H1N1/2009 influenza. Aims of this study were to analyse antibody responses and the factors associated with high antibody titres in a cohort of children with naturally acquired A/H1N1/2009 influenza infection confirmed by reverse-transcriptase polymerase chain reaction (RT-PCR).

**Results:**

Demographic, clinical and virologic data were collected from 69 otherwise healthy children with pandemic A/H1N1/2009 influenza (27 females, mean age ± SD: 5.01 ± 4.55 years). Their antibody levels against pandemic A/H1N1/2009 and seasonal A/H1N1 influenza viruses were evaluated by measuring hemagglutination-inhibiting antibodies using standard assays. Sixty-four patients (92.8%) with pandemic A/H1N1/2009 influenza had A/H1N1/2009 antibody levels of ≥40, whereas only 28/69 (40.6%) were seroprotected against seasonal A/H1N1 influenza virus. Those who were seroprotected against seasonal A/H1N1 virus were significantly older, significantly more often hospitalised, had a diagnosis of pneumonia significantly more frequently, and were significantly more often treated with oseltamivir than those who were not seroprotected (*p *< 0.05). The children with the most severe disease (assessed on the basis of a need for hospitalisation and a diagnosis of pneumonia) had the highest antibody response against pandemic A/H1N1/2009 influenza virus.

**Conclusions:**

Otherwise healthy children seem to show seroprotective antibody titres after natural infection with pandemic A/H1N1/2009 influenza virus. The strength of the immune response seems to be related to the severity of the disease, but not to previous seasonal A/H1N1 influenza immunity.

## Background

An A/H1N1 quadruple reassortant influenza virus (A/H1N1/2009) of swine origin has recently arisen from a subtype A/H1N1 influenza virus that was already endemic in humans. It caused a pandemic [1], with a very high disease burden among children and young adults: up to 50% showed signs of infection, against 10% of the adult population [2,3]. Severe disease and hospitalisations were also associated with younger age groups [4,5].

Serological analyses of pre-pandemic serum samples showed that a number of adult and elderly subjects had higher levels of cross-reactive A/H1N1/2009 antibodies than young adults and children (the older the patient, the higher the levels) [2,6,7]. It has been suggested that the age-related differences in the frequency and severity of pandemic influenza infection were due to multiple exposures to older viruses with similar B cell epitopes, and the conservation of T cell epitopes between the seasonal A/H1N1 and pandemic A/H1N1/2009 viruses [8,9].

However, very few pediatric data are available, and little is known about the proportion of pediatric pandemic A/H1N1/2009 influenza cases showing seroconversion, the magnitude of this seroconversion, or the factors influencing the antibody levels evoked by the pandemic A/H1N1/2009 influenza virus.

The aim of this study was to contribute towards filling these gaps by analysing antibody responses and the factors associated with high antibody titres in a cohort of children with naturally acquired pandemic A/H1N1/2009 influenza infection confirmed by reverse-transcriptase polymerase chain reaction (RT-PCR).

## Results

Sixty-nine of the 101 initially enrolled children (68.3%; 27 females; mean age 5.01 ± 4.55 years) were positive for pandemic A/H1N1/2009 influenza virus assessed by RT-PCR and were included in the final analysis. Table 1 shows their demographic and clinical characteristics. Sixty-four (92.8%) had pandemic A/H1N1/2009 antibody levels of ≥40, whereas only 28 (40.6%) were seroprotected against seasonal A/H1N1 virus. Those who were seroprotected against seasonal A/H1N1 influenza virus were significantly older, significantly more often hospitalised, had a diagnosis of pneumonia significantly more frequently, and were significantly more often treated with oseltamivir than those who were not seroprotected. There were no differences in the geometric mean titres (GMTs) of pandemic A/H1N1/2009 antibodies or viral load between the children who were seroprotected against seasonal A/H1N1 influenza virus and those who were not.

**Table 1:** Demographic and clinical characteristics of children with pandemic A/H1N1/2009 influenza infection

Variables	Seroprotection against seasonal A/H1N1 influenza virus (n = 28)	Absence of seroprotection against seasonal A/H1N1 influenza virus (n = 41)
Age		
<2 yrs	2 (7.1)*	18 (43.9)
2-5 yrs	8 (28.6)	13 (31.7)
>5 yrs	18 (64.3)*	10 (24.4)
Mean age ± SD	5.03 ± 4.38*	2.38 ± 4.66
Gender, females	9 (32.1)	18 (43.9)
Previous influenza vaccination	0 (0.0)	0 (0.0)
Hospitalisation	9 (32.1)*	4 (9.8)
Diagnosis		
Pneumonia	15 (53.6)*	10 (24.4)
Upper respiratory tract Infection	13 (46.4)*	31 (75.6)
		
Antibodies against pandemic A/H1N1/2009 influenza virus		
≥40	27 (96.4)	37 (90.2)
GMT	163.33	211.46
Viral load, log_10 _cp/mL		
Mean value ± SD	7.71 ± 1.69	7.70 ± 1.64
Treated with oseltamivir	15 (53.6)*	10 (24.4)

*GMT *geometric mean titres, *SD *standard deviation. Numbers with percentages in parenthesis. *p <0.05 vs absence of seroprotection against seasonal A/H1N1 influenza virus

Table 2 shows that high pandemic A/H1N1/2009 antibody titres (≥160, detected in 26 patients) were not associated with age, gender, viral load or oseltamivir treatment. Univariate analysis showed that the patients with the highest pandemic A/H1N1/2009 antibody titres were significantly less often seroprotected against seasonal A/H1N1 influenza virus, but this association was not confirmed by the multivariate analysis. On the contrary, the children with the most severe disease (as evaluated on the basis of the need for hospitalisation and a diagnosis of pneumonia) had the highest antibody response to pandemic A/H1N1/2009 influenza virus at both univariate and multivariate analysis.

**Table 2:** Association between high antibody titres against pandemic A/H1N1/2009 influenza virus (≥160) and other variables

Variables	Univariate OR (95% CI)	Multivariate OR adjusted for all variables (95% CI)
Age (yrs)		
<median value (4.5)	1	1
≥ median value	0.48 (0.18-1.28)	0.60 (0.18-1.98)
Gender	1	1
Female		
Male	0.57 (0.21-1.53)	0.54 (0.14-2.00)
Hospitalisation		
No	1	1
Yes	3.38 (1.49-10.61)	4.43 (1.55-14.16)
Pneumonia		
No	1	1
Yes	4.54 (1.57-13.14)	5.92 (1.66-21.18)
Previous immunity against seasonal A/H1N1 influenza virus		
<40	1	1
≥40	0.27 (0.09-0.81)	0.70 (0.17-2.93)
Viral load, log_10 _cp/mL		
<median value (8.2)	1	1
≥ median value	1.79 (0.67-4.78)	1.78 (0.55-5.77)
Oseltamivir therapy		
No	1	1
Yes	1.13 (0.55-4.43)	1.19 (0.61-4.76)

*CI *confidence interval, *OR *odds ratio

## Discussion

The findings of this study clearly show that otherwise healthy children can produce a protective immune response when they are infected by the recent pandemic A/H1N1/2009 influenza virus. They are in line with those of authors studying seasonal influenza viruses [10,11], and confirm that most children, including those aged less than two years, seem to have an immune system that can efficiently face influenza virus infection. More than 90% of the convalescent children in our population had a pandemic A/H1N1/2009 antibody level of ≥40 about four weeks after the onset of their illness. Both the seroconversion rate and the magnitude of antibody responses of our healthy children were similar to those reported by others studying convalescent sera obtained from older patients [2,12-14].

However, despite these similarities, some of our findings are quite different from those found in adult and elderly subjects: those previously exposed to seasonal A/H1N1 influenza viruses experienced significantly less severe influenza [2-5], whereas the rates of hospitalisation and a diagnosis of pneumonia were significantly higher in our children with protective antibodies against seasonal A/H1N1 influenza virus than in those without. Moreover, on the basis of what has been demonstrated in experimental animals, we expected to find a stronger immune response to the pandemic A/H1N1/2009 influenza virus in children who had previously been affected by seasonal A/H1N1 influenza. It has been found that the previous exposure of ferrets to a contemporary seasonal A/H1N1 influenza virus is capable of priming a greater antibody response to a subsequent dose of a non-adjuvanted monovalent pandemic A/H1N1/2009 influenza vaccine [15], but we found no significant difference in pandemic A/H1N1/2009 antibody levels between our children who were positive for antibodies against seasonal A/H1N1 infection and those who were negative. It is possible that both these findings can be explained by non-immunological factors. The lack of any correlation between previous immunity against seasonal A/H1N1/2009 influenza virus and severity of influenza disease can simply be the consequence of an age effect. Influenza is per se more severe in younger patients (i.e., those less exposed to seasonal viruses) and these characteristics have been clearly demonstrated also for the recent pandemic [4,5]. Moreover, data collected in experimental animals cannot be easily compared with those found in humans because these are very little homogeneous by age and sex and the global sample can be potentially affected by a selection bias. However, both these findings can be explained by means of the so-called "antigenic sin phenomenon", according to which the relative absence of exposure to influenza virus variants (as is usual in otherwise healthy children who have not received an influenza vaccination) may promote viral spread, whereas multiple exposures to variant viruses (as is usual in older people or subjects repeatedly vaccinated against seasonal influenza) may engender greater immune protection because of the immune system's capacity for cross-reactivity [16]. This hypothesis is consistent with the findings of Laurie *et al*. [17], who showed that ferrets previously exposed to repeated infections due to seasonal influenza A viruses were significantly more protected against pandemic A/H1N1/2009 influenza virus than those exposed to a single infection.

The antibody responses to pandemic A/H1N1/2009 influenza virus in our children were not influenced by age, gender, previous immunity against seasonal A/H1N1 influenza virus, viral load or the use of oseltamivir, but were significantly greater in the patients with the most severe disease. Our viral load data conflict with those reported by Hung *et al*. [14], who found a strict correlation between convalescent neutralising antibody titres and viral load. However, it is difficult to evaluate the effect of viral load on the characteristics of influenza because viral load peaks 1-2 days after symptom onset [14,18], frequently before the patients attend an Emergency Room and their nasopharyngeal secretions are collected.

Our findings concerning oseltamivir are in line with those of Hung *et al*. [14], and what has been demonstrated in subjects suffering from seasonal influenza [18,19]. These findings clearly show that antiviral treatment does not affect the subsequent humoral immune response to viral infection, and so it is unlikely to be associated with a higher rate of recurrence or reinfection.

Finally, our data regarding the greater immune response of children with more severe disease are quite similar to those found in adults by Mak *et al*. [12], and further support the conclusion that the immune system of healthy children seems capable of facing pandemic influenza infection.

## Conclusions

Despite collected in a relatively small number of patients, our findings suggest that the immune response of children after natural infection with pandemic A/H1N1/2009 influenza virus evokes adequate seroprotection. The strength of the immune response seems to be related to the severity of the disease, but not to previous immunity against seasonal A/H1N1 influenza. However, further studies are needed to determine how long efficacious antibody levels last and whether patients with the poorest immune response may be affected by new episodes during the same influenza season.

## Methods

### Study patients

The study involved subjects aged less than 15 years without any underlying chronic severe disease who attended the Emergency Room or were hospitalised during one of the two weeks of the peak period of pandemic A/H1N1v influenza in Italy (9-15 November 2009) because of an influenza-like illness as defined by the Italian Ministry of Health http://http//www.ministerosalute.it. This definition is an acute respiratory disease of sudden onset and with fever (an axillary temperature of >38°C), accompanied by at least one of the general symptoms of headache, generalised malaise, a feverish sensation (sweating and chills) or asthenia, and by at least one of the respiratory symptoms of cough, pharyngodynia or nasal congestion.

The exclusion criteria were chronic diseases increasing the risk of complications of viral respiratory infections, including premature birth; chronic disorders of the pulmonary or cardiovascular systems, including asthma; chronic metabolic diseases, including diabetes mellitus; neoplasia; kidney or liver dysfunction; hemoglobinopathies; immunosuppression; diseases requiring long-term aspirin therapy; and genetic or neurological disorders. The enrolled patients' demographic characteristics and medical history were systematically recorded using standardised written questionnaires as previously described [20,21] and, after a complete physical examination, they were classified into disease groups on the basis of signs and/or symptoms using well-established criteria [22]. Upon enrolment, a nasopharyngeal swab for the diagnosis of pandemic A/H1N1/2009 influenza infection was collected from all of the children, as was a blood sample for the evaluation of antibody levels against the pandemic A/H1N1/2009 and the seasonal A/H1N1 viruses circulating in the previous two years.

The participants who were positive for pandemic A/H1N1/2009 influenza infection were asked to return for a final visit four weeks (28 ± 2 days) after enrolment, during which clinical data were collected and a further blood sample was drawn to determine anti-A/H1N1/2009 antibody levels.

The study was approved by the Institutional Review Board of the Fondazione IRCCS Ca' Granda Ospedale Maggiore Policlinico, Milan, Italy, and was carried out in the Department of Maternal and Pediatric Sciences of the University of Milan. The written informed consent of a parent or legal guardian was required, and the older children were asked to give their assent.

### Laboratory assays

Each nasopharyngeal sample was obtained using a pernasal flocked swab, and stored in a tube of UTM-RT (Kit Cat. No. 360c, Copan Italia, Brescia, Italy). Viral RNA was extracted from the swabs by means of a Nuclisens EasyMAG automated extraction system (Biomeriéux, Craponne, France). All of the samples were tested for the detection and characterisation of pandemic A/H1N1/2009 influenza virus following the WHO/CDC protocol [23]. A plasmid containing the corresponding target viral sequence (kindly provided by the Molecular Virology Unit, Fondazione IRCCS Policlinico San Matteo, Pavia, Italy) was used to quantify viral load. Ten-fold plasmid serial dilutions ranging from 5 to 5 × 10^7 ^input copies were prepared to generate calibration curves, and run in parallel with the tested samples.

The cycle threshold (Ct) values of each dilution were measured in duplicate and plotted against the logarithm of their initial quantities. The copy numbers in each clinical sample were derived from the regression line. The quantitative results were expressed as RNA copy number/mL of nasopharyngeal swab following data multiplication by 50. In order to evaluate reproducibility, intra-assay and interassay standard deviations (SDs) and coefficients of variation were calculated for each standard concentration within and between the individual PCR runs.

A/H1N1/2009 and seasonal A/H1N1 antibody levels were evaluated by measuring hemagglutination-inhibiting (HI) antibodies using standard assays [24]. The serum samples were tested in duplicate at an initial dilution of 1:10, and the HI antibody titres were expressed as the reciprocal of the highest serum dilution that completely inhibited hemagglutination. GMTs were computed by assigning a titre of 5 for the samples in which antibodies were undetectable. A subject was considered seroprotected if his/her HI titre was ≥40.

### Statistical analysis

The continuous variables are presented as mean values ± standard deviation (SD), and the categorical variables as numbers and percentages. The continuous data were analysed using a non-parametric test (i.e. two-sided Wilcoxon rank sum test) as they were not normally distributed (based on the Shapiro-Wilk statistic); the categorical data were analysed using contingency tables and the chi-squared or Fisher's test, as appropriate.

The univariate and multivariate odds ratios (ORs) of high antibody titres against pandemic A/H1N1v virus (i.e. ≥160), and their 95% confidence intervals (CI), were derived using unconditional multiple logistic regression models. The multivariate model was calculated using terms for age and gender, hospitalisation, a diagnosis of pneumonia, immunity against A/H1N1 seasonal influenza virus, A/H1N1/2009 viral load, and oseltamivir therapy.

## Abbreviations

(CI): Confidence intervals; (GMTs): Geometric mean titres; (HI): Hemagglutination-inhibiting; (ORs): Odds ratios; (RT-PCR): Reverse-transcriptase polymerase chain reaction; (SD): Standard deviation.

## Competing interests

The authors declare that they have no competing interests.

## Authors' contributions

SE and NP designed the study and co-wrote the manuscript. CD, AS and FB carried out the RT-PCR and quantification of viral load. CT and IP visited the patients. FT collected the swabs. CG performed the statistical analysis. All authors read and approved the final manuscript.
